# Core competencies of rural practice for medical students of government‐sponsored medical education programs in Taiwan: The students' perspective

**DOI:** 10.1002/jgf2.70045

**Published:** 2025-07-14

**Authors:** Shih‐Ming Li, Hang‐Rui Zhang, Hsin‐Yun Chang, Cheng‐Loong Liang, Wang‐Huei Sheng, Tsung‐Ying Chen, Wen‐Yuan Lin, Zih‐Jie Sun, Jin‐Shang Wu

**Affiliations:** ^1^ Department of Counseling and Clinical Psychology National Dong Hwa University Hualien Taiwan; ^2^ Department of Family Medicine, National Cheng Kung University Hospital, College of Medicine National Cheng Kung University Tainan Taiwan; ^3^ Institute of Allied Health Sciences, College of Medicine National Cheng Kung University Tainan Taiwan; ^4^ School of Medicine I‐Shou University Kaohsiung Taiwan; ^5^ School of Medicine National Taiwan University College of Medicine Taipei City Taiwan; ^6^ Department of Anesthesiology, Hualien Tzu Chi Hospital Buddhist Tzu Chi Medical Foundation/School of Medicine, Tzu Chi University Hualien Taiwan; ^7^ Department of Family Medicine, China Medical University Hospital China Medical University Taichung Taiwan; ^8^ Department of Family Medicine, National Cheng Kung University Hospital, Dou‐Liu Branch, College of Medicine National Cheng Kung University Yunlin Taiwan; ^9^ Department of Family Medicine, National Cheng Kung University, College of Medicine National Cheng Kung University Tainan Taiwan

**Keywords:** ABC model, core competency, rural area

## Abstract

**Background:**

The shortage of rural physicians remains a public health concern. Placing medical students in rural areas and exposing them to rural physicians as models may enhance physician retention in rural areas. The purpose of this study was to explore the core competencies of medical students for rural practice and develop a framework of such competencies.

**Methods:**

A three‐stage mixed method was used to develop the core competencies of medical students who will engage in rural practice. In the exploring stage, four physicians completed in‐depth interviews; eight students participated in the focus group to identify the core competencies. In the construct stage, two students were interviewed individually and four students as a group to construct the framework. Finally, the competencies were validated by experts in the verification stage. In addition, the analytic hierarchy process was used.

**Results:**

After thematic analysis, three themes—*adaptation*, *befriending*, *and career*—emerged as the ABC model for rural practice. The framework of competencies for rural practice was established with the ABC model and validated by the analytic hierarchy process (AHP).

**Conclusions:**

Although some competencies vary across professional stages, the ABC model—adaptation, befriending, and career—is the competencies essential for medical students preparing to practice in rural areas.

## 1 INTRODUCTION

The shortage of rural physicians remains a public health concern, with 19% of the US population but only 11% of all physicians residing in rural areas.^1^ In rural areas, the doctor/patient ratio ranges from 68 to 100,000, compared unfavorably to the 84 to 100,000 ratio in cities.^2^ In Taiwan, about 16% of the population lives in rural areas.^3^ However, it is established that rural areas have a significantly lower physician density than urban areas, with many rural communities having critically low physician numbers below WHO standards, although a precise percentage figure for physicians practicing in rural Taiwan is not available.^4^ Family physicians are the primary doctors in rural areas. Family medicine students who have received or experienced training in rural medicine are 5–6 times more likely to serve in rural areas in the future. However, <10% of all medical students receive training in rural medicine.^5^ In Taiwan, the retention rate of doctors at their original medical institution in rural areas is <20%, and that of obstetrics and gynecology practitioners is only 6.12%.^6^ The Optimization of Rural Health Care Improvement Plan indicates that most of the medical facilities in rural areas are run by the public sector. The retention of physicians is a pressing concern for rural medicine in Taiwan, which is becoming increasingly urbanized. Some social issues, such as poverty, chronic disease, poor mental health, and addiction, are challenges for the rural population.^7^ A major barrier to health‐care access for these populations is the well‐documented shortage of health‐care providers.^8^ The shortage of rural physicians may be exacerbated by declining student interest in primary care careers and a declining proportion of medical students pursuing internships in rural areas, despite being historically more likely to provide care in rural areas.^9^

A survey of national graduates found that only 272 of 6483 respondents (response rate, 4.2%) were trained in a rural residency.^8^ In the rural area, four themes are related to the patient experience: navigating the rural environment, navigating the health‐care system, financing chronic disease management, and rural life.^10^ The factors that encourage interest in rural employment are a welcoming community, partner employment, family located in the rural area, and an outdoor lifestyle.^11^ Recently, clinical training and education in rural areas have been found to encourage health professionals to work permanently in rural locations.^12^ A meta‐analysis found that a rural background and rural clinical experiences may help predict physicians' intention to practice rural medicine.^12^ A review article revealed that factors associated with the retention of physicians in rural areas include commitment to a rural curriculum and rotations.^13^ Placing medical students in rural areas and exposing them to rural physicians as models may enhance physician retention in rural areas.^14^ To ensure the supply of highly qualified physicians in medically underserved areas (rural communities) and recruit physicians to less attractive medical specialties, two types of government‐sponsored medical education programs were introduced in Taiwan.^15^, ^16^

The Accreditation Council for Graduate Medical Education (ACGME) and the American Board of Medical Specialties (ABMS) have developed a framework that identifies six core competencies in medical education that can be applied in any medical practice. Still, rural practice presents challenges that may demand some specific competencies. In family medicine, meeting the demands of the rural community is the basic goal of medical training.^17^ Eight competencies—including adaptability, agency and courage, collaboration and community responsiveness, comprehensiveness, integrity, abundance in the face of scarcity and limit, reflective practice, and resilience—have been suggested for rural practice.^18^ The concept of six competency domains is also observed in rural areas.^18^ The framework of the competencies can be modified for specific areas or training situations, and there may be some common competencies across situations, such as the six core competencies in ACGME and eight competencies in rural contexts.

In a rural setting, physicians must treat and stabilize patients with injuries and illnesses from a broad range of clinical conditions.^19^ They must engage more thriving needs such as excitement and interactive value based on the functional theory.^20^ Experienced practitioners can offer rural patients continual care,^21^ but working in rural areas can be professionally stunting.^22^ Rural longitudinal integrated clerkships are a solution for rural medicine, and connectivity was the key to retention, including current practice, future practice, and social network.^23^ Supporting trainees in developing their professional identity as rural physicians through rural‐specific competencies and positive experiences in rural areas could improve one's retention in rural areas.^24^ In the rural area, some competencies will be enhanced, like adaptation in the rural area,^18^ enhancing the social network^20^ and professional identity in the rural area.^19^, ^20^ In this study, we explored the core competencies of medical students for engaging in a rural setting by adopting a three‐stage mixed method. The purpose of this study was to explore the core competencies of medical students for rural practice and develop a framework for such competencies.

## 2 METHODS

### 2.1 Design

We conducted in‐depth interviews, a focus group, an expert group, and the analytic hierarchy process (AHP) to construct competencies in this mixed‐methods research from November 2022 to June 2023. Data were collected from multiple sources to explore competencies relevant to a rural setting.^25^ The author employed a snowball strategy to survey medical students and rural physicians. This study was approved by the Research Ethics Committee of National Cheng Kung University Hospital (approval number: B‐ER‐111‐088).

### 2.2 Three stage mixed method

A three‐stage mixed method was used to develop the core competencies of medical students who will engage in rural practice.

#### 2.2.1 Stage 1: Exploring the core competencies for rural practice

We conducted in‐depth interviews with rural physicians and a focus group with medical students who will be government‐sponsored physicians after their graduation to develop the preliminary framework of the core competencies for rural practice and identify related learning issues.

There were four rural physicians who participated in in‐depth interviews, and they all worked in rural medical institutions, two of them on Taiwan's main island and two on outlying islands. The first one (A) was a family physician who has practiced in the rural area for a long time in Taitung, Taiwan. Following the recommendation of A, B practiced medicine in Hualien, Taiwan. Then C and D were introduced by B. An emergency physician (C) and a surgeon (D) work on the outlying islands of Taiwan. These four rural physicians have worked for a long time in eastern Taiwan.

The seven students in the focus group were all medical students who intended to join rural practices and have attended teaching hospitals in southern Taiwan. The first author has more than 10 years of experience in qualitative research, and he conducted the interview and the focus group.

##### In‐depth interview

Four physicians in the rural area of Taiwan were invited for in‐depth interviews. Two male physicians were from the rural area, and two male physicians were from the outlying islands. These physicians discussed the core competencies for rural practice with the first author. We then invited the students to confirm whether the findings fit their experience in the focus group. Each interview lasted for 60–90 min. The interview questions were as follows:

1. Tell me about your experience serving in rural areas.
2. What do you think are the competencies required for rural services?
3. What training content do medical students need to be ready to work in rural areas?
4. What competencies do you need for the cultivation of professionalism in medical students who will serve in rural areas in the future?
5. What is your approach to current rural service training?
6. What suggestions do you have for current medical education?

##### Focus group

Seven medical students from grade 1 to grade 3 in Southern Taiwan were invited to participate in a focus group led by the first author. Four students were male, and three were female. Their ages were between 20 and 24. They shared their perspectives on the core competencies and training needs for rural practice. The questions were as follows:

1. Please share your experience of service in rural areas.
2. What competencies do medical students need to develop for work in rural areas?
3. In rural service training, what curricular topics are important?
4. In your rural service experience, what themes have you learned and what abilities have you developed?
5. What types of reforms do you think current medical education requires to facilitate future rural practice?
6. Personally, what factors make you willing to serve in rural areas?

#### 2.2.2 Stage 2: Constructing the framework of competencies for rural practice

We conducted in‐depth interviews with two medical students and a focus group with four medical students to confirm the framework of the core competencies for rural practice by the first author. All medical students at this stage had experience serving in rural areas.

##### Focus group

Four male medical students from grade 3 to grade 5 were invited to participate in a focus group by the first author. They shared their perspectives on the core competencies and training needs for rural practice. The questions were as follows:

1. What, according to you, are the competencies required for rural practice?
2. For the framework of core competencies (please refer to the stage 1 results), which competencies are important, and which need to be modified?
3. In your opinion, what needs to be corrected and supplemented in the competency framework?
4. In rural medical training, are there any other content to be added?

##### In‐depth interview

In‐depth interviews were conducted by two male medical students in grade 3 from northern Taiwan. The medical students with rural experience identified the core competencies for rural practice and confirmed whether they fit their experience. Each interview lasted for 60 min. The interview questionnaire comprised the following questions:

1. Please share your experience of service in rural areas.
2. What do you think are the competencies required for rural practice?
3. For the framework of core competencies (please refer to stage 1 results), which competencies are important, and which need to be modified?
4. In your opinion, what needs to be corrected and supplemented in the competency framework?

#### 2.2.3 Stage 3: Validating the framework of competencies for rural practice

We conducted expert group sessions twice with five physicians who have had rural experience, and we also conducted AHP with 15 medical students who intend to practice in rural areas. This was performed to validate the framework of competencies for rural practice.

##### Expert group

An expert group was convened to verify the core competencies and framework identified through stages 1 and 2. We invited five experts who had experience in designing training materials for rural practice. These experts—three Family Medicine, one Internal Medicine, and one Anesthesiologist—have engaged in rural medicine for more than 10 years. Two were from northern Taiwan, two from eastern Taiwan, and the last one was from southern Taiwan. These experts have extensive experience in teaching and providing medical care and involvement in designing and administering training programs in rural medicine. Two unstructured group meetings were held to verify the core competencies and the framework of competencies for rural practice.

##### The analytic hierarchy process (AHP)

AHP involves weighting factors to determine relative values of the core competencies, which were derived from the preliminary results from stage 1 and stage 2. According to the guidelines for expert decision‐making, 15–30 homogeneous experts are ideal, and data reliability increases when >10 experts are included.^26^ Before factor weights were calculated on the basis of the AHP survey, the matrix constructed for each respondent was checked for intrapersonal consistency. The closer the maximum eigenvalue (*λ*) is to the number of factors, the more consistent and noncontradictory the subjective judgments of the decision‐makers are. A consistency ratio of ≤0.1 indicates a satisfactory degree of matrix consistency. Judgments are considered to be consistent if the consistency index (CI) value is <0.1.^27^ Thus, a CI value of <0.1 could be the criterion for selecting a sample for the AHP.^28^

**FIGURE 1:**
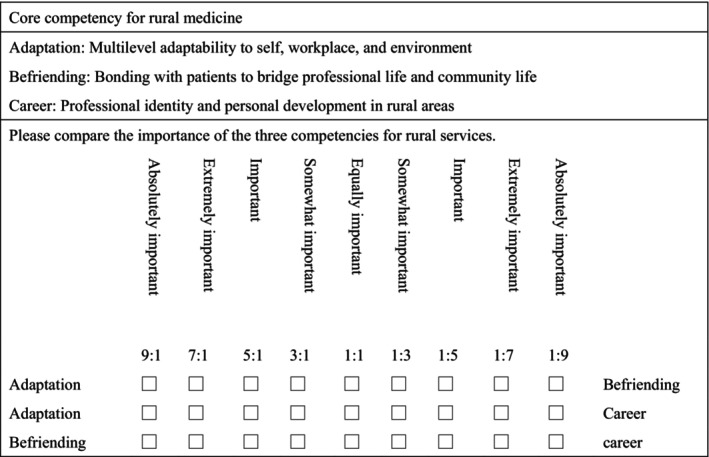
AHP questionnaire for three core competencies.

A total of 15 medical students completed the questionnaire (Figure 1). Of the students, 12 were women and 3 were men. The mean age was approximately 22.6 ± 2.44 years.

##### Qualitative data analysis

A six‐step model of thematic analysis^29^ was adopted to formulate the core competencies. The steps of the thematic analysis were as follows:

1. Gaining familiarity with the data: Read the text carefully, become familiar with the context of the interview, and separate paragraphs according to their meanings on a word‐by‐word basis.
2. Generating initial codes: Extract and encode key phrases according to the meaning of each paragraph.
3. Identifying themes: Summarize the theme of each paragraph based on key phrases.
4. Reviewing themes: Group‐related themes into common themes.
5. Defining and naming themes: Name the core themes according to the research goal.
6. Reporting the results: Consolidate the results into graphs for review by the interviewees.

Each interview was transcribed and analyzed by two researchers. The findings were verified by a review team comprising two family medicine physicians. The paragraph data were coded in the format of “code‐ID‐line.” Codes I and G represented the in‐depth interview and focus group, respectively. The numbers denoted the identification numbers of the interviewees. The lines indicated the number of lines contained in a paragraph in the transcript. For example, “I‐A‐3” indicated the interview data from the third line of the in‐depth interview transcript of interviewee A.

A coding system was carefully developed; peer review debriefs were conducted; and regular reflexive team meetings were held to avoid researcher bias. The adaptation competency involves three levels: self, workplace, and environment coding as A1, A2, and A3. The befriending competency involves connecting to patients, professionals, and the community as B1, B2, andB3. The career competency involves identity, passion, and knowledge as C1, C2, and C3. The coding system is presented in Table 1.

**TABLE 1:** The competency framework of rural medicine.

Core competency	Sub‐competencies	Code	Quotes
Adaptation	Self‐adjustment	A1 Self	“[You] can find something that makes you happy spiritually. Imagine that you've just arrived in a new environment. If you are not happy with the material aspect of life, try exploring the spiritual side of things for happiness.” (G‐K‐041)
	Being familiar with workplace	A2 Work	“When I first started practicing, I couldn't find enough nurses for the work, so I had to do a lot by myself…I used to work almost 14 h a day.” (I‐B‐047)
	Integration into local life	A3 Life	“[Learning about] the locals' day‐to‐day routines, I started to adjust my own [to ensure that I am available] at the time they finish field work.” (I‐B‐053)
Befriending	Establishing doctor–patient relationship	B1 Patient	“There are fewer patients now, and the service quality is much better. I have more time to learn about the lives of my patients, discuss their symptoms, and understand the improvements they are expecting.” (G‐N‐100)
	Professional co‐operation	B2 Professional	“We work to maintain good connections with hospitals. The patients should be made aware of this effort, rather than keeping it to ourselves, because it's reassuring to them.” (I‐A‐095)
	Linkage community	B3 Community	“Caring for highly respected members of the community is actually a very important part of building a relationship [with the community].” (I‐A‐092)
Career	Professional identity	C1 Identity	“In some of the situations, [you] still need that sense of belonging. But I think people recognize our work and need our work, so there is no problem with our work.” (I‐B‐037)
	Keeping passion in rural area	C2 Passion	“I also felt like a passer‐by myself at first, but sometimes, as time goes by, I feel like I've become part of the community. So, I think of ways to improve the medical environment here.” (I‐C‐055)
	Application of knowledge	C3 Knowledge	“[It's about] whether you're willing to learn new things. It'll be better if they're willing to try new things beyond their medical practice.” (I‐B‐120)

##### Quantitative data analysis

IBM Statistical Product and Service Solutions (SPSS) (version 14.0) was used for descriptive statistics. Power‐choice (version 3.5) for AHP was used for checking the λ_max_, the consistency index (CI), the consistency ratio (CR), and weighting.

### 2.3 Data saturation

The data were analyzed case‐by‐case. The interview data of interviewees A and B and focus group in stage 1 were analyzed to extract the three themes for core competencies. Then, the three themes were confirmed with the data from interviewees C and D, and no more themes were found. In advance, the framework of core competency had been constructed in the focus group at stage 2 and confirmed by in‐depth interview and expert group at stage 3.

### 2.4 Reflexivity

Reflexivity is a set of continual, collaborative, and multifaceted practices through which researchers self‐consciously appraise and evaluate how their subjectivity and context influence the research process.^25^ In the process of data collection and analysis, the reflexivity of the researcher provides a more objective interpretation of the data.^30^ Throughout the qualitative study phase, reflexivity was maintained by the first author (SM‐L) through regular research meetings with physicians who had rural practice experience. The remainder of the research team (a qualitative researcher, a community medical physician, a medical educator, and a clinical psychologist) provided the multiple opinions for self, interpersonal, methodological, and contextual reflexivity.

## 3 RESULTS

Following our three‐stage mix‐method, there were three findings discussed in the following sections.

### 3.1 The ABC model for the rural context

The thematic analysis revealed the following three themes—adaptation, befriending, and career—emerged as the ABC model for rural practice.

Adaptation is the process of changing oneself to suit different conditions, such as the workplace or a rural area. When working in a rural area, some physicians adapt well to the environment and enjoy their life; however, others find it difficult and leave. Adaptation is a competency through which one can engage with the rural community and learn the rural lifestyle. When physicians adapt to rural areas, they can fit themselves into the local life and serve for a long time.

I believe in people's ability to adapt, so I just got used to the rural life. (G‐J‐032)

I struggled to adapt at first, but slowly I realized that that's just who they are, and there are actually reasons for their behavior. (I‐C‐048)

[Learning about] the locals' day‐to‐day routines, I started to adjust my own [to ensure that I am available] at the time they finish field work. (I‐B‐053)

In the rural setting, befriending means that a practitioner must connect with the community and bond with patients, their families, and all community members. There are three levels of connection: one is to bond with patients, another is to connect with other people and professionals in one's network, and still another is to engage with the community.

The ability to build an interpersonal network is also a competency. As I said earlier, you need to be sensitive, connect with them, and earn their trust. In circumstances where there aren't enough staff members, the only thing we can do is build a healthier community. (I‐A‐039)

We also provide mobile clinic services that go to their homes. This brings us closer to their lives and helps us better understand their lives, not only their medical conditions but also the challenges they face in their lives. (I‐C‐032)

At that time, medical services were actually something that could easily bring people together. So, we built—we assisted them in building—a volunteer network to support the community. (I‐A‐038)

Career refers to professional identity and passion in a rural setting. When serving in rural areas, physicians encounter many challenges, such as limitations related to salary, education, family, and low opportunities for professional development. For their career, the physicians will keep learning professional knowledge for the rural challenge by themselves. When physician commit their career in rural areas, they will have a sense of belonging to the community and spend a long time in the rural area. On the other hand, when they do not commit their career in rural areas, they will leave.

As for continuing education, staying in a medical center allows us to see and treat patients with more complex conditions, and that's what I enjoy doing. Another thing is, as I've mentioned, the resources. There are more accessible and available resources for self‐improvement. Next, if I'm to start a family and have children, the kids' education can be a problem because the teaching resources may not be as readily available in rural areas. It's going to be me living away from my family, yeah. But, if it's in the city center, [the kids may have] better school options or peers. (G‐O‐081)

First of all, professional knowledge is certainly important. What have you learned in your training? Second, we organize small‐scale seminars for discussions. Those junior to us are better at computers, so they can even use video calls to connect with those in [the main island of] Taiwan, which allows us to take lessons and earn our credits from here. (I‐B‐022)

### 3.2 The validation of the ABC model from AHP

**FIGURE 2:**
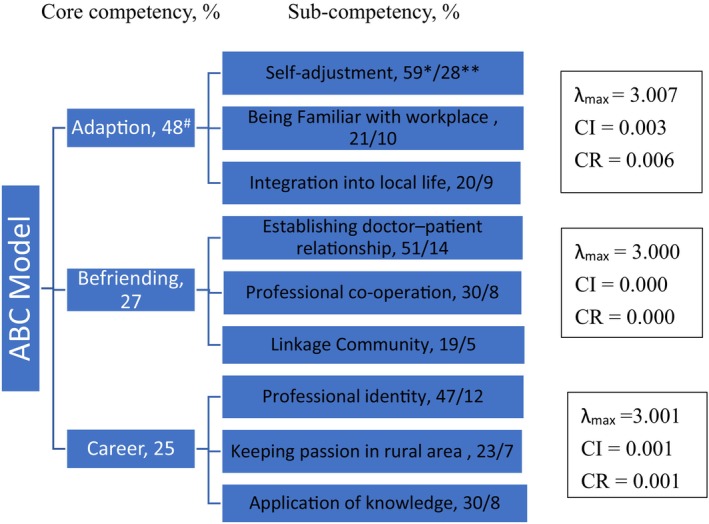
The importance of core competencies and their sub‐competencies of the ABC model. ^#^Weight of Adaptation, Befriend, and Career core competencies by a total of 100%. *Weight of the three sub‐competencies of each competency by a total of 100%. **Weight of the nine sub‐competencies by a total of 100%.

Based on the competency framework, an AHP questionnaire was established in two expert meetings. Then, 15 medical students completed the AHP questionnaire. We excluded six samples with a high consistency index (CI > 0.1) in the three core competencies, and nine samples were used in the final analysis (λ_max_ = 3.022; CI = 0.011; CR = 0.020 for ABC model). Regarding the importance of the three core competencies, adaptation weighted 47.5%, befriending 27.3%, and career 25.2%. Under each core competency, the importance of the sub‐competency was from 19% to 59%. Among the nine sub‐competencies, the most important ones were adjustment (27.8%), bonding/relationships (14.0%), identity (11.9%), and workplace familiarity (10.2%; Figure 2).

### 3.3 The relationship between the ABC model and ACGME's six competencies

**FIGURE 3:**
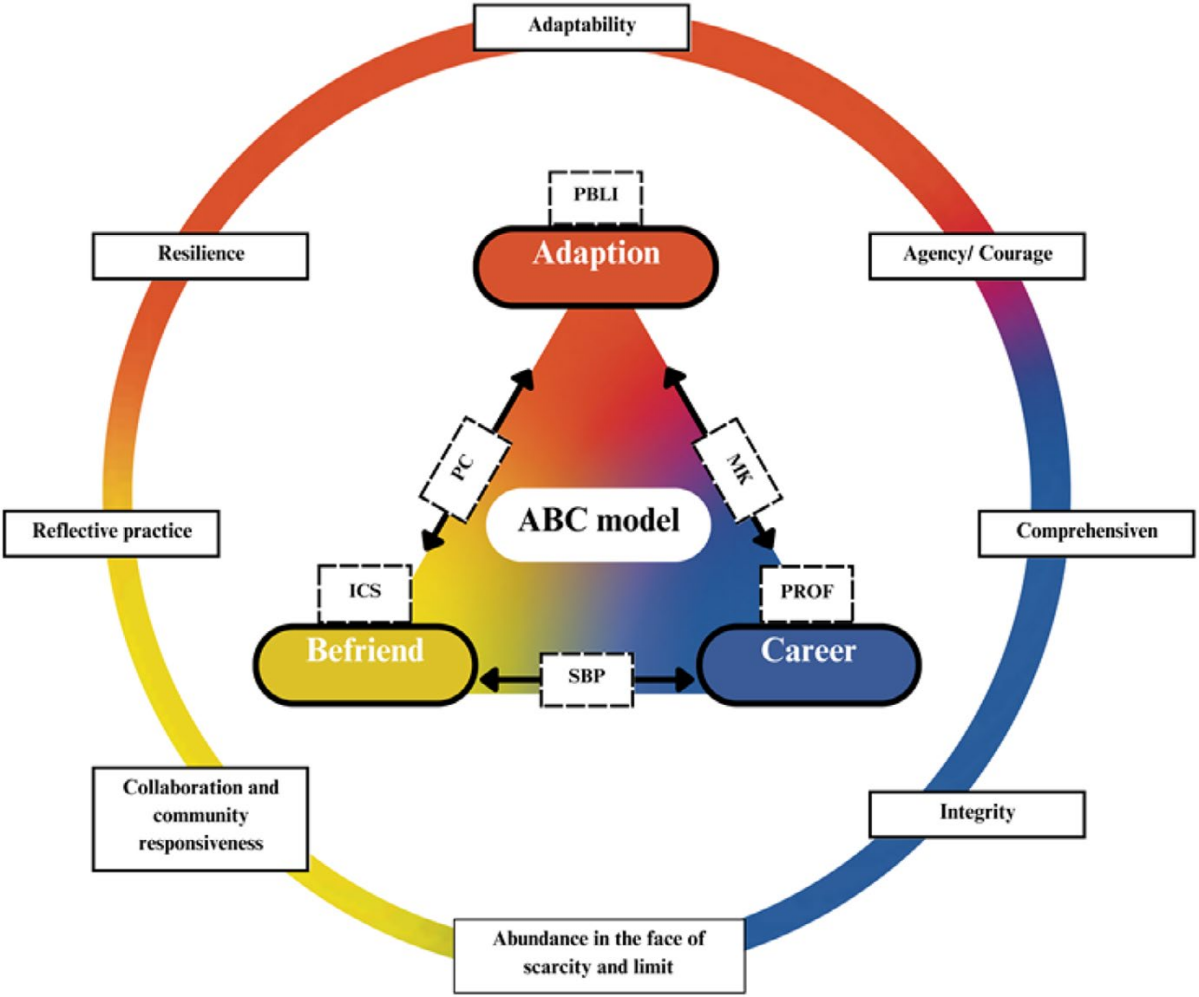
The relationship of ABC model with ACGME six competencies (square in dotted line) and rural eight competencies (square in solid line).

The possible relationships between the core competencies of the ABC model (adaptation, befriending, and career) and those of ACGME's six competencies are shown as Figure 3. Patient care (PC) is an appropriate treatment of health problems and health promotion. In the rural context, the physician will adapt practice to community needs and befriend the patient to provide rural practice. Medical knowledge (MK) is the application of physician knowledge to patient care and could be the combination of adaptation and career. Systems‐based practice (SBP) is the ability to call effectively on other resources in the system to provide optimal health care. In the rural area, SBP must connect local life and engage the local community to practice in the system as the befriending plus career. Practice‐based learning and improvement (PBLI) is the ability to provide patient care with the best current scientific evidence and to continuously improve patient care based on constant self‐evaluation and life‐long learning.^25^ As adaptation is related to the modified care in rural areas, rural physicians have to keep learning to adapt to the community. Interpersonal and communication skills (ICS) is the effective exchange of information and collaboration with patients, their families, and health professionals, which is the same as the befriending. Professionalism (PROF) is highly related to the career, and physicians will commit to their careers with high professionalism in rural areas.

Especially for the older people, even going up the stairs can be difficult, and there are many older people who live alone. This is probably why the doctors start to bring medications and record books along, and visit older people one after another. (I‐D‐041)

We will organize some small seminars to discuss together…, and then we can take classes here to get credits. (I‐B‐022)

We also provide mobile clinic services at their homes. This brings us closer to their lives and makes us understand their lives more, not only their medical conditions but also the challenges they face in their lives. (I‐C‐032)

I also felt like a passer‐by myself at first, but sometimes, as time goes by, I feel like I've become one of the community. So, I think of ways to improve the medical environment here. (I‐C‐55)

I think of it as working in a new environment. Don't reject the local customs. Try participating in the local activities and adapting to the local community's customs. (G‐E‐037)

To ensure good teamwork, it's important to communicate, be understanding of others, and adapt to others' opinions. (I‐D‐022)

## 4 DISCUSSION

### 4.1 Finding

Based on the three‐stage mixed method, three core competencies for rural practice were identified in this study, and they were adaptation, befriending, and career, which we refer to as the ABC model. The medical students who intended to rural areas need the competency to adapt to the rural area,^18^ suitable personality traits or skills like befriending the community,^17^, ^31^ and more career commitment to rural than urban.^15^ After AHP, with paired comparison to determine the relative weights of the ABC model components with regard to their importance, adaptation weighted 47.5%, befriending 27.3%, and career 25.2%. We also identified the sub‐competencies of each core competency. For total sub‐competencies, the first three important sub‐competencies were self‐adjustment, establishing *doctor–patient* relationships, and professional identity with their weights of 28%, 14%, and 12%, respectively. Based on the three core competencies and the nine sub‐competencies, the framework of rural medicine competencies was developed for medical students (Table 1). As the findings from the intention to rural practice, self‐directedness and cooperativeness were the important personality traits. Those were related to the competencies, such as career and befriending,^31^ and adaptation was the core competency to enter rural areas. Those findings^18^, ^32^ were compatible with our finding that the ABC model is the basic component for medical students who intend to practice in rural areas.

Rural practice presents challenges and opportunities requiring unique training.^18^ Based on the ABC model and eight competencies of rural practice, the adaptation of the ABC model was related to the first core competency of rural context, which is adaptability. Integrity may not only involve the integration into local life of adaptation but also in professional cooperation of befriending. Then, collaboration and community responsiveness may be echoed in befriending. Previously, when physicians commit to their careers in rural areas, they may need agency/courage, comprehensiveness, reflective practice, and resilience. However, they must accept the abundance in the face of scarcity and limits in rural practice.^18^ Medical students may have a better chance of strengthening their competence in this area through their social identification with their background culture and the network of people they build in accepting policies and learning.^33^ With the start of the ABC model, the students could be trained to nourish the ACGME's six core competencies and enhance the eight‐domain capabilities in a rural context.^18^

Health professionals in rural communities have challenges and unique opportunities.^24^ It can be rewarding to become close to a community through adaptation and connect with patients at a deep level. However, limited resources can be a turn‐off to practicing rurally. Students can be attracted for training in rural settings through educational innovations, advancing an interprofessional model of care (such as befriending), addressing burnout, promoting well‐being (such as adaptation), and elevating the integral role of professionals (such as career).^24^ A community‐based clinical immersion program may encourage medical students to join a rural practice.^32^ Physicians wishing to practice in rural areas eventually face challenges that may require additional knowledge, skills, attitudes, values, and other professional attributes. Adaptability is the core competency for rural contexts.^18^ According to the findings of the ABC model, adaptation is the most important competency for medical students who intend to practice in rural areas. For rural students, befriending means that they must stay connected with the place and cultivate a special connection with its people. Finally, medical students consider their career in rural areas to be a big decision and a self‐professional identity. As the low retention rate of doctors at their original medical institution in rural areas,^4^ the career, also called professionalism or professional identity, will be important for long‐term rural area practice.

**FIGURE 4:**
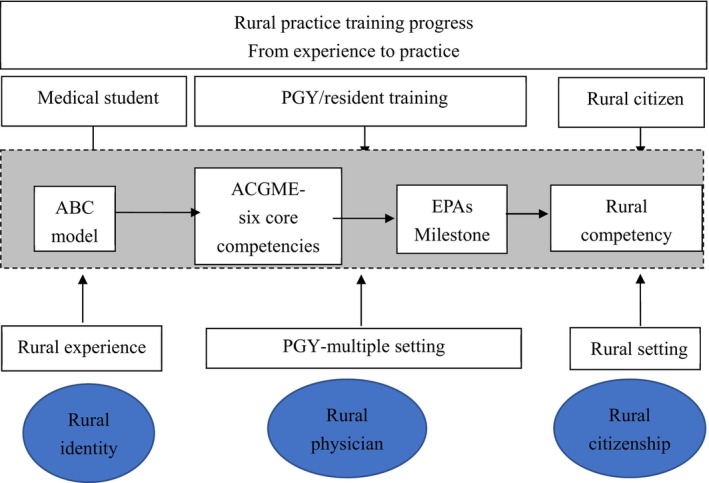
The proposed process of rural practice training from medical student to resident and rural citizen.

“I also felt like a passer‐by myself at first, but sometimes, as time goes by, I feel like I've become part of the community.” (I‐C‐055). The physician (I) had transformed from outsider to a member in the community, and the connectivity could enhance retention.^34^ Based on the ABC model, adaptation, befriending, and career were the key competencies for rural practice. If medical students can develop these three abilities through outdoor activities or rural experience, this will be beneficial to the continued service in rural areas in the future. Regarding continual training in rural settings,^19^ community enrichment and the ability to evolve to meet patient demands are the key concerns for rural residency programs.^17^ Community enrichment focuses on how physicians integrate into the community through buy‐in, professional identity, and other factors, such as adaptation, befriending, and career. The ability to evolve to meet patient demands is focused mainly on education, such as training in community medicine. Training in multiple settings is beneficial for developing the six ACGME core competencies.^33^ Rural practice involves developing a rural identity, developing a rural physician identity, initiating rural practice, solidifying the rural physician identity, identifying as a rural physician, and remaining in rural practice.^24^ Medical students should cultivate the three core competencies—adaptation, befriending, and career—by gaining some rural experience and then, in the postgraduate year (PGY) program, acquire all six core competencies.^35^ In the rural setting, physicians can enhance their competencies^18^ specific to the rural setting. In rural communities, place‐based education focuses on relational connectedness in both work and the broader social environment, which promotes student engagement in the community.^24^ Some rural community programs encourage students or physicians to serve in such settings.^5^ Following the ABC model, rural medical training could be proposed as Figure 4, but it needs further study.

### 4.2 Limitations

We recruited a convenience sample of rural physicians and medical students who intend to practice in rural areas; nonetheless, our data achieved thematic saturation. The snowball method is used to collect in‐depth information through the personal connections of the existing research sample, but it is limited by the existing social network. In this study, most of the family physicians and other specialties will be included in future studies. Another limitation is the generalizability of our findings because of the cultural or social differences that influence the education system among countries.^19^ Nevertheless, the primary goal of this study was reached, and we identified the three core competencies of the ABC model for medical students intending to practice in rural areas. In this study, we focus on Taiwan's rural area under the collectivism culture which emphasizes the unity of the group or community and found the adaptation, befriending, and career as core competencies. In some cultural contexts, such as overseas‐born health professionals working in rural areas, understanding and respecting local health beliefs including complementary and alternative medicine are important for effective community integration,^34^ which is similar to adaptation and befriending in our findings. Nevertheless, the primary goal of this study was reached, and we identified the three core competencies of the ABC model for medical students intending to practice in rural areas. In the future, more studies like longitudinal studies are needed for the ABC model's impact on retention or related training programs on rural medicine.

### 4.3 Implication

Extracurricular activities could enhance the three competencies: adaptation, befriending, and career. For example, outdoor courses can enhance rural experience for adaptation; teamwork and community‐engaging programs can train befriending; and value exploring can be beneficial for career development.^34^ Based on the ABC model, adaptation, befriending, and career were the core competencies for rural practice. If medical students can develop these three abilities through outdoor activities or rural experience, it will be beneficial for them to continue their service in rural areas in the future.

### 4.4 Conclusion

In conclusion, although some competencies vary across professional stages, the ABC model is the core competency essential for medical students preparing to practice in rural areas. Formal national rural training may be an essential part of the broader system for rural workforce development and enhance the competencies for the rural areas.^36^, ^37^

## AUTHOR CONTRIBUTIONS

**Shih‐Ming Li:** Conceptualization; methodology; formal analysis; writing – original draft; writing – review and editing; software; investigation. **Hang‐Rui Zhang:** Data curation; investigation; formal analysis. **Hsin‐Yun Chang:** Data curation; investigation. **Cheng‐Loong Liang:** Resources; supervision. **Wang‐Huei Sheng:** Resources; supervision. **Tsung‐Ying Chen:** Resources; supervision. **Wen‐Yuan Lin:** Resources; supervision. **Zih‐Jie Sun:** Conceptualization; methodology; resources; writing – review and editing; supervision. **Jin‐Shang Wu:** Writing – original draft; writing – review and editing; conceptualization; resources; supervision; project administration.

## CONFLICT OF INTEREST STATEMENT

The authors have stated explicitly that there are no conflicts of interest in connection with this article.

## ETHICS STATEMENT

Ethics approval statement: This study was approved by the Research Ethics Committee of National Cheng Kung University Hospital (approval number: B‐ER‐111‐088).

Patient consent statement: None.

Clinical trial registration: None.
